# Utility of Non-albumin Proteinuria for the Assessment of the Severity of Tubulointerstitial Inflammation in Lupus Nephritis Patients

**DOI:** 10.7759/cureus.77608

**Published:** 2025-01-18

**Authors:** Nur Jahan, Muhammad Rafiqul Alam, A. K. M Shahidur Rahman, S. M. Remin Rafi, S. M. Shamsuzzaman, Noureen Amin, Mamun Chowdhury Raju

**Affiliations:** 1 Department of Nephrology, National Institute of Kidney Diseases and Urology (NIKDU), Dhaka, BGD; 2 Department of Nephrology, Bangabandhu Sheikh Mujib Medical University (BSMMU), Dhaka, BGD; 3 Department of Nephrology, Dhaka Medical College and Hospital, Dhaka, BGD

**Keywords:** glomerulonephritis (gn), lupus nephritis (ln), non-albumin proteinuria (nap), systemic lupus erythematosus (sle), tubulointerstitial inflammation (ti)

## Abstract

Background

Lupus nephritis (LN) is the most common severe manifestation of systemic lupus erythematosus (SLE) that can involve all kidney components. The International Society of Nephrology/Renal Pathology Society (ISN/RPS) 2003 lupus nephritis classification only focuses on glomerular involvement, although tubulointerstitial inflammation (TI) is a better predictor of renal failure.

Objective

To determine the association of non-albumin proteinuria (NAP) and severity of tubulointerstitial inflammation in lupus nephritis patients.

Methods

This cross-sectional study was carried out in the Department of Nephrology of Bangabandhu Sheikh Mujib Medical University (BSMMU), Dhaka, Bangladesh. A total of eighty (80) LN patients were enrolled in this study. Their urinary protein-to-creatinine ratio (uPCR), urinary albumin-to-creatinine ratio (uACR), renal biopsy, and relevant routine investigations were done accordingly. Urinary non-albumin proteinuria (uNAP) was measured by subtracting uACR from uPCR. Data were analyzed and compared by statistical tests.

Results

Among 80 lupus nephritis patients, 39 (48.8%) had no-to-mild tubulointerstitial inflammation (TI), whereas 41 (51.2%) had moderate-to-severe tubulointerstitial inflammation. Proliferative glomerulonephritis (GN) (class III/class IV) was observed in 48 patients (60%), while non-proliferative GN (class II/V) was present in 32 patients (40%). In the logistic regression analysis, non-albumin proteinuria (uNAP) was found to be associated with moderate-to-severe tubulointerstitial inflammation (OR: 3.166, 95% CI: 1.145-8.757, p=0.026). The calculated cutoff value for uNAP was 887, which corresponds to a sensitivity of 96.7% and specificity of 76.0% (p<0.001).

Conclusion

Non-albumin proteinuria is associated with the severity of tubulointerstitial inflammation in lupus nephritis. Therefore, assessing non-albumin proteinuria can offer clinically valuable insights into the management of lupus nephritis.

## Introduction

Systemic lupus erythematosus (SLE) is a multisystem, autoimmune disorder of the connective tissue. One of the most common and severe manifestations is lupus nephritis (LN), which affects approximately 60% of SLE patients [1]. The current classification for lupus nephritis patterns was established by the International Society of Nephrology/Renal Pathology Society (ISN/RPS) in 2003, solely based on glomerular pathology [2]. However, the pathological alterations in lupus nephritis are not restricted to the glomerulus alone but can involve all structures of the kidney, including the glomeruli, tubules, interstitium, and blood vessels [3]. Among these pathological modifications, tubulointerstitial (TI) involvement of the kidneys is another well-known but less commonly highlighted abnormality in SLE [4,5]. Moreover, several studies have indicated that the degree of tubulointerstitial involvement, as opposed to the type of glomerular lesion, offers more accurate predictive insights into the long-term renal effects of lupus nephritis [1,6,7]. The absence of a correlation between the ISN/RPS classification and the severity of tubulointerstitial involvement implies that distinct mechanisms underlie glomerular and tubulointerstitial pathologies [8]. According to earlier studies, systemic autoimmunity is the main cause of glomerulonephritis (GN), although localized immunological mechanisms within the kidney may also have an impact on severe tubulointerstitial damage [9,10]. Therefore, the degree of tubulointerstitial injury is not correlated with systemic markers like complement levels and high anti-dsDNA antibodies, which are associated with glomerular disorders [1,7]. The mechanisms and categories of urinary proteins differ between glomerular and tubulointerstitial disorders. Glomerular proteinuria results from the glomerular filtration barrier being damaged, which makes it easier for plasma proteins that are typically prohibited from entering the glomerular filtrate to cross the barrier [11]. As a result, in cases of glomerular pathologies, urinary proteins primarily consist of higher molecular weight proteins, predominantly albumin [12]. On the other hand, in cases of tubulointerstitial disorders, urinary protein excretion occurs because tubules are unable to reabsorb filtered proteins due to tubular dysfunction [13]. Thus, the prevailing urinary proteins associated with tubulointerstitial pathologies consist of non-albumin proteins (NAPs) like α1-microglobulin, retinol-binding protein, and β2-microglobulin [12]. Hence, taking into consideration the dissimilarity in urinary protein compositions between glomerular and tubulointerstitial conditions, earlier studies have proposed using the urinary albumin-to-creatinine ratio (uACR)/urinary total protein-to-creatinine ratio (uPCR) for identifying the source of proteinuria [14,15]. But approximately 38% to 66% of SLE patients display both glomerulonephritis (GN) and tubulointerstitial (TI) involvement in kidney biopsies [16-19]. This contrasts with other renal disorders that primarily or exclusively involve one of these compartments, like membranous nephropathy [8]. Therefore, in cases of lupus nephritis where both GN and tubulointerstitial involvement coexist, the utilization of NAP appears more effective than using uACR/uPCR to assess the severity of tubulointerstitial inflammation [8]. Furthermore, non-albumin proteinuria (NAP), a measure of tubulointerstitial damage, is highly related to poor renal response after six months of immunosuppressive treatment [8,20]. Earlier research offers strong indications that commonly accessible serological indicators do not exhibit any connection with tubulointerstitial involvement, emphasizing the necessity for discovering new biomarkers and tailored treatments. Hence, the purpose of this study was to determine the relationship between non-albumin proteinuria and the degree of tubulointerstitial inflammation, as this relationship may provide important clinical information for lupus nephritis patients.

## Materials and methods

Study design

This cross-sectional observational study was conducted at the Department of Nephrology, Bangabandhu Sheikh Mujib Medical University (BSMMU), Dhaka, Bangladesh. The study received approval from the institutional review board (IRB approval number: BSMMU/2019/13978), BSMMU, Dhaka, Bangladesh.

Study population

A total of 80 patients who were diagnosed as having lupus nephritis (LN) in the Department of Nephrology, Bangabandhu Sheikh Mujib Medical University (BSMMU), Dhaka, Bangladesh, between October 2019 and September 2020 were enrolled by purposive sampling technique following selection criteria. Diagnosed adult (age>18 years) cases of SLE with lupus nephritis, according to 2019 ACR classification criteria for SLE [21], were included in this study. Patients with active infections, autoimmune diseases other than SLE, any types of malignancy, patients with other causes of glomerulopathies, and pregnant/lactating women were excluded from the study.

Study procedure

After the selection of the study population, voluntary informed written consent was taken from every patient with an explanation of the procedure and purpose of the study. The natural history, pathophysiology, relevant investigations, current treatment options, and consequences of lupus nephritis were explained to them. A detailed case history with clinical findings was recorded, and their anthropometric measurement was done. A fresh urine sample of each patient was collected and sent for urinary total protein, urinary albumin, and urinary creatinine estimation. All other hematological/biochemical tests of blood and urine, such as urine for routine microscopic examination (R/M/E), complete blood count (CBC), serum creatinine, C-reactive protein (CRP), serum complements level (C3, C4), anti-nuclear antibody (ANA), and anti-double standard DNA (anti-dsDNA), were measured accordingly. Urinary albumin-to-creatinine ratio (uACR) was estimated by dividing spot urinary albumin by spot urinary creatinine and was expressed as mg/g. Urinary protein-to-creatinine ratio (uPCR) was measured by dividing urinary protein by urinary creatinine and was expressed as mg/dl, which was converted to mg/g by multiplying with 1000. Urinary non-albumin proteinuria (uNAP) was quantified by deducting uACR from uPCR (uNAP = uPCR - uACR).

Tubulointerstitial inflammation (TI)

After doing all necessary investigations, a renal biopsy was done after obtaining consent of the patient. Then renal tissues were sent for histopathological and direct immunofluorescence examination. The International Society of Nephrology (ISN)/Renal Pathology Society (RPS) classification criteria were used for renal histopathology. Two pre-determined pathologists without prior knowledge of the clinical outcome categorized the tubulointerstitial inflammation (TI) severity, which was semi-quantitatively scored based on the percentage of the non-scarred cortical area involved by mononuclear cell infiltrate. The scores ranged from 0 to 4, which corresponded to no (0%), minimal (<10%), mild (10-25%), moderate (26-50%), and severe (>50%) inflammatory cell infiltration in the tubulointerstitium [8].

Histological classes of lupus nephritis on renal biopsy

Histological classes of lupus nephritis were categorized as class I to class VI [2] and grouped as proliferative (class III and IV) and non-proliferative (class II and V) during analysis.

Data analysis

All data were recorded systematically in a preformed data collection form. A descriptive analysis was performed for the demographic, clinical, and histopathological characteristics. Continuous data were analyzed using arithmetic mean as a reliable measure of central tendency and standard deviation (SD) as a measure of dispersion. Categorical data were summarized using frequency and percentages. Statistical analyses were conducted using the IBM SPSS Statistics for Windows, Version 26 (Released 2019; IBM Corp., Armonk, New York, United States). For comparisons among different groups, the Mann-Whitney U test and unpaired t-test were used for continuous variables, and the chi-squared test for categorical variables. Pearson’s correlation and Spearman’s correlation analyses were used to observe the correlation between the variables. To predict the parameters linked to moderate to severe tubulointerstitial (TI) damage, a logistic regression analysis was conducted. Statistical significance was defined as a p-value of less than 0.05.

## Results

A total of 80 lupus nephritis patients were included in the study; their mean age was 28.0±8.7 years. The ratio of females to males was 7:1, with 70 (87.5%) being female and 10 (12.5%) being male. Most of them were in the third decade (<30 years); 46 (57.5%) patients had nephrotic range proteinuria, 46 (57.5%) had hematuria, 39 (48.8%) patients were hypertensive, and 37 (46.3%) patients had raised serum creatinine (Table 1).

**Table 1:** Basic characteristics of the study patients (N=80) BMI: body mass index; anti-dsDNA: anti-double standard DNA; uPCR: urinary protein-to-creatinine ratio

Variables	Number of patients (n)	Percentage (%)
Age group (years)		
<30	54	67.5
30-40	21	26.3
>40	5	6.3
Gender		
Male	10	12.5
Female	70	87.5
BMI (kg/m²)		
Underweight	13	16.3
Normal weight	59	73.8
Overweight	8	10.0
Anaemia		
Mild	58	72.5
Moderate	13	16.3
Absent	9	11.3
Hypertension	39	48.8
Haematuria	46	57.5
uPCR (≥ 3500 mg/g)	46	57.5
Raised serum creatinine	37	46.3
Positive anti-dsDNA	58	72.5
Low C3	52	65.0
Low C4	34	42.5

The two TI severity groups (no to mild TI and moderate to severe TI) showed significant differences in non-albumin proteinuria (NAP), while the traditional markers (C3, C4, anti-dsDNA) did not show significant differences. These findings did not exhibit similarity between the two histological categories (proliferative/non-proliferative). Those with ISN/RPS class III or IV proliferative nephritis had a higher rate of moderate to severe TI.

Table 2 shows light microscopic and DIF findings of renal biopsy specimens. According to the ISN/RPS classification, most of the patients were class IV (40%). The distribution of TI severity was as follows: minimal, mild, and moderate inflammation in 4 (5%), 35 (43.8%), and 41 (51.2%) patients, respectively (Table 2).

**Table 2:** Histological features of the study patients (N=80) ISN/RPS: International Society of Nephrology/Renal Pathology Society

Histological features	Number of patients (n)	Percentage (%)
ISN/RPS Classification		
Class II	15	18.8
Class III	16	20.0
Class IV	32	40.0
Class V	17	21.3
Tubulointerstitial inflammation		
No inflammation	0	0.0
Minimal	4	5.0
Mild	35	43.8
Moderate	41	51.2
Severe	0	0.0
Tubular atrophy		
No to mild	77	96.3
Moderate to severe	3	3.8
Interstitial fibrosis		
No to mild	77	96.3
Moderate to severe	3	3.8

Table 3 shows comparisons among the patients with different TI severities. The moderate to severe TI group had a higher uPCR value (p=0.014), higher non-albumin proteinuria (uPCR-uACR) (p<0.001) and raised serum creatinine (p=0.042) than the no to mild group. But there was no statistically significant relationship between TI severity and C3, C4, or anti-dsDNA titre (p>0.05 for each measure).

**Table 3:** Comparison of laboratory parameters between TI severity groups (N=80) Data were expressed as mean (±SD); p-value obtained by the unpaired t-test/Mann-Whitney U test uPCR: urinary protein-to-creatinine ratio; uACR: urinary albumin-to-creatinine ratio; anti-dsDNA: anti-double standard DNA

Variables	TI Severity		Z/t- value	p-value
	No to mild (n=39)	Moderate to severe (n=41)		
uACR (mg/g)	2376.3 ± 2445.1	2432.1 ± 2322.9	0.114	0.432
uPCR (mg/g)	3176.8 ± 2868.3	4576.3 ± 3310.3	2.103	0.014
uACR/uPCR	0.63 ± 0.21	0.54 ± 0.24	-1.781	0.075
uPCR - uACR (mg/g)	840.2 ± 785.2	2122.6 ± 1713.6	4. 545	<0.001
Serum creatinine(mg/dl)	1.05 ± 0.34	1.22 ± 0.39	2.048	0.042
C3 (g/L)	0.54 ± 0.38	0.51 ± 0.45	-0.358	0.415
C4 (g/L)	0.48 ± 0.75	0.32 ± 0.58	-1.105	0.483
Anti-dsDNA (IU/ml)	157.0 ± 196.5	145.3 ± 236.2	-0.239	0.257

Table 4 demonstrates that patients with ISN/RPS Class III and IV had significant moderate-to-severe TI (p<0.001). No significant differences were observed in tubular atrophy or interstitial fibrosis between the groups (p>0.05). 

**Table 4:** Comparison of histological findings between TI severity groups (N=80) P-value obtained by the chi-square test TI: tubulointerstitial inflammation; ISN/RPS: International Society of Nephrology/Renal Pathology Society

Variables	TI Severity		χ2 value	p-value
	No to mild (n=39)	Moderate to severe (n=41)		
ISN/RPS Classification				
III and IV, n (%)	16 (41.0)	32 (78.0)	13.11	<0.001
II and V, n (%)	23 (59.0)	9 (22.0)		
Tubular atrophy				
No-to-mild, n (%)	39 (100.0)	38 (92.7)	2.97	0.085
Moderate-to-severe, n (%)	0 (0.0)	3 (7.3)		
Interstitial fibrosis				
No-to-mild, n (%)	39 (100.0)	38 (92.7)	2.96	0.085
Moderate-to-severe, n (%)	0 (0.0)	3 (7.3)		

Table 5 demonstrates a comparison between the different histological types according to the ISN/RPS classification. Patients with proliferative GN (class III/IV) had lower C3 and C4 (p=0.041, p<0.001), higher titers of anti-dsDNA antibodies (p=0.015) and higher uPCR (p=0.004), uACR (p=0.018) compared to nonproliferative GN (class II/V). There was no significant difference in non-albumin proteinuria (uPCR-uACR) between the proliferative and nonproliferative GN groups (Table 5).

**Table 5:** Comparison of laboratory parameters between proliferative and non-proliferative groups (N=80) Data is expressed as mean (SD), P-value obtained by the unpaired t-test/Mann-Whitney U test GN: glomerulonephritis; uPCR: urinary protein-to-creatinine ratio; uACR: urinary albumin-to-creatinine ratio; TI: tubulointerstitial inflammation; anti-dsDNA: anti-double standard DNA

Variables	Proliferative GN (n=32)	Non-proliferative GN (n=48)	Z/t- value	p-value
uACR (mg/g)	2921.1±2774.9	1651.11±1414.26	-2.784	0.018
uPCR (mg/g)	4578.8±3519.9	2573.4±1645.9	-3.443	0.004
uACR/uPCR	0.57±0.23	0.63±0.20	1.543	0.236
uPCR - uACR (mg/g)	1923.8±1179.7	1638.96±757.38	-1.451	0.107
Serum creatinine(mg/dl)	1.19±0.39	1.02±0.32	-2. 312	0.004
C3 (g/L)	0.45±0.39	0.64±0.39	2.342	0.041
C4 (g/L)	0.10±0.06	0.87±0.91	4.865	<0.001
Anti-dsDNA (IU/ml)	200.0±261.2	85.1±56.9	-2.946	0.015

Table 6 shows that TI severity differed significantly between proliferative and non-proliferative groups (p<0.001). No significant differences were observed for tubular atrophy or interstitial fibrosis (p>0.05) (Table 6).

**Table 6:** Comparison of histological findings between proliferative (III and IV) and non-proliferative (II and V) groups (N=80) P-value obtained by the chi-square test ISN/RPS: International Society of Nephrology/Renal Pathology Society

Variables	ISN/RPS Classification		χ2 value	p-value
	III and IV (n=48)	II and V (n=32)		
TI Severity				
No-to-mild, n (%)	16 (33.3)	23 (71.9)	11.42	<0.001
Moderate-to-severe, n (%)	32 (66.7)	9 (28.1)		
Tubular atrophy				
No-to-mild, n (%)	45 (93.8)	32 (100.0)	2.08	0.149
Moderate-to-severe, n (%)	3 (6.3)	0 (0.0)		
Interstitial fibrosis				
No-to-mild, n (%)	46 (95.8)	31 (96.9)	0.58	0.810
Moderate-to-severe, n (%)	2 (4.2)	1 (3.1)		

The variables linked to moderate-to-severe TI in lupus nephritis were assessed using a multivariate logistic regression analysis. Notably, uPCR-uACR was significantly associated with moderate-to-severe TI (odds ratio (OR) 3.00, 95% confidence interval (95% CI) 0.239-7.124, p=0.039) (Table 7).

**Table 7:** Multivariate logistic regression analysis to predict the risk factors of moderate-to-severe TI (N=80) uPCR: urinary protein-to-creatinine ratio; uACR: urinary albumin-to-creatinine ratio; TI: tubulointerstitial inflammation; anti-dsDNA: anti-double standard DNA

Variables	p-value	OR	95% CI	
			Lower	Upper
Age	0.821	1.211	0.229	6.404
Female	0.824	1.007	0.944	1.076
uACR	0.615	1.001	0.417	1.006
uPCR	0.018	1.999	0.304	4.021
uPCR – uACR	0.039	3.000	0.239	7.124
uACR/uPCR	0.803	1.221	0.240	2.441
Serum creatinine	0.017	1.351	0.177	3.605
C3	0.695	0.751	0.180	3.138
C4	0.651	1.267	0.455	3.523
Anti-dsDNA	0.739	1.000	0.998	1.003

**Figure 1:**
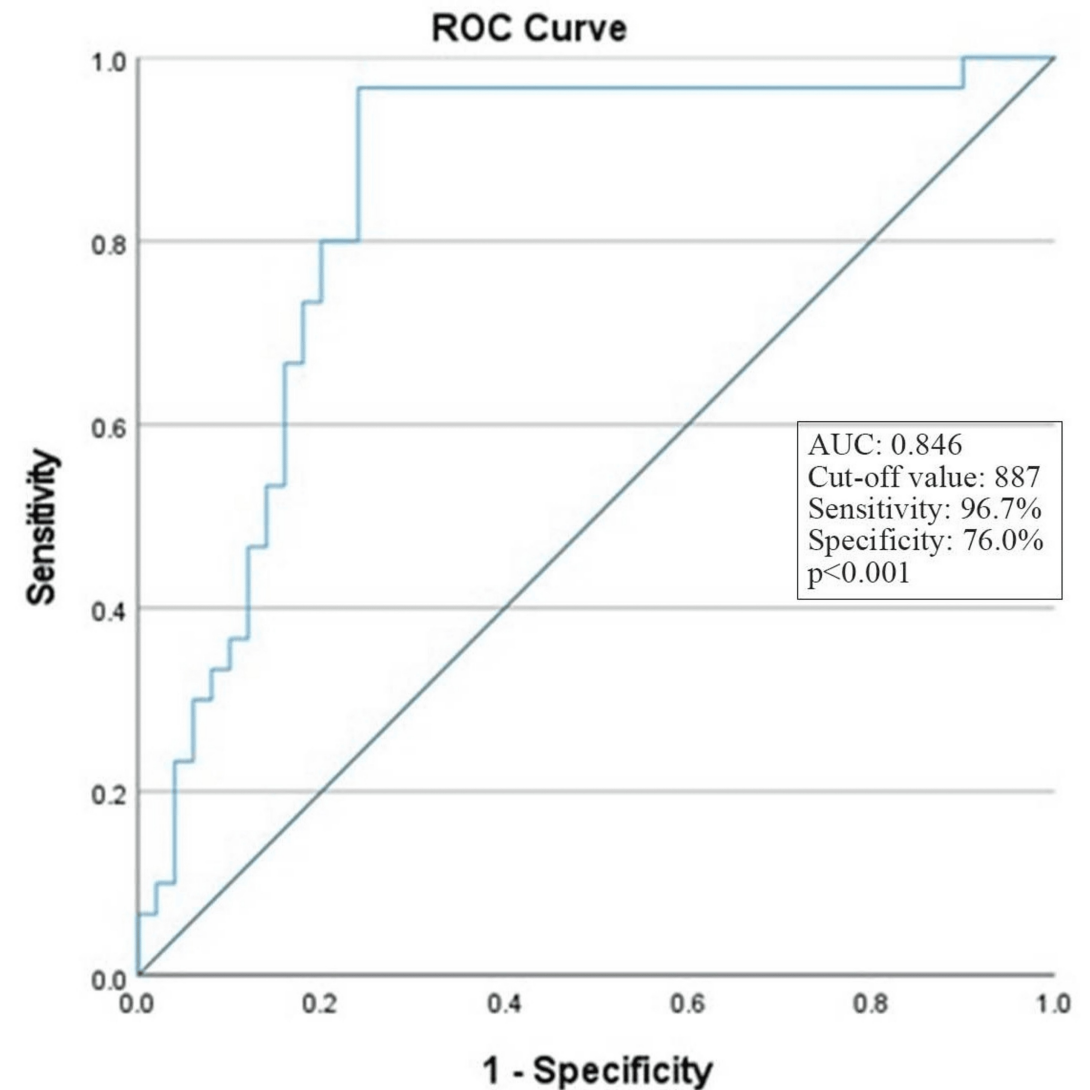
ROC curve analysis to measure the best cutoff value of uPCR-uACR of TI severity ROC: receiver operating characteristic; uPCR: urinary protein-to-creatinine ratio; uACR: urinary albumin-to-creatinine ratio; TI: tubulointerstitial inflammation; AUC: area under the curve

The receiver operating characteristic (ROC) curve analysis was conducted to determine the optimal cutoff value for tubulointerstitial (TI) severity based on the analysis of uNAP. The area under the curve (AUC) was 0.846, signifying a strong discriminatory ability of the uNAP in predicting TI severity. The cut-off value for uNAP was 887. This cut-off value corresponds to a sensitivity of 96.7% and specificity of 76.0%. The p-value was <0.001, indicating statistical significance (Figure 1).

## Discussion

Patients with lupus nephritis often have tubulointerstitial renal disease [22]. It has been reported that 69.4% of lupus nephritis patients have tubulointerstitial inflammation [23]. Previous studies established that the widely accepted 2003 ISN/RPS classification of LN could not predict treatment response and progression to end-stage renal disease (ESRD) as it relies exclusively on glomerular pathology [2,24]. Assessing renal tubulointerstitial injury in LN is highly significant for determining the extent of kidney damage, guiding treatment decisions, and predicting the outlook for the condition [6,7]. Given this context, a readily accessible parameter that accurately reflects the severity of TI is desperately needed to meet the requirements effectively. This cross-sectional study explores the association of non-albumin proteinuria with the severity of tubulointerstitial inflammation in lupus nephritis patients. A total of 80 patients aged 18 years or above were enrolled in this study. The mean age of the study patients was 28.0 years. It was documented that lupus nephritis is more frequent in younger patients aged less than 45 years. Parikh et al. [24] also suggested that SLE predominantly affects women of childbearing age [24]. The demographic findings were almost consistent with this study. It was observed that most lupus patients were female (87.5%). This female predominance is attributed primarily to sex hormones [25]. According to Christou et al. [25], sexual dimorphism in SLE is largely caused by single-nucleotide polymorphisms (SNPs) in genes and sex hormones.

This study found a significant correlation (p<0.05) between moderate to severe TI and the levels of uPCR, uPCR-uACR, and serum creatinine. Unlike previous studies that proposed using uACR/uPCR as a method for identifying the source of proteinuria [14,15], the current study did not find a significant correlation between the severity of TI and uACR/uPCR (OR 1.221, 95% CI 0.240-2.441, p=0.803). It was noted that lupus nephritis cases often exhibit pathological alterations in both the glomeruli and the tubulointerstitium. The co-occurrence of glomerular disease increases the variability of urine albumin excretion, which may explain why uACR/uPCR is not as reliable as uPCR-uACR in determining the severity of TI [8]. In this study, it was noted that certain cases exhibited severe glomerular damage alongside mild tubulointerstitial changes, and conversely, there were cases with pronounced tubulointerstitial changes but relatively minor glomerular injury, mirroring the findings of previous studies [1,3,8,26]. Therefore, glomerulonephritis and tubulointerstitial nephritis can occur independently. Furthermore, recent research indicates that glomerulonephritis results from systemic autoimmunity, while severe tubulointerstitial inflammation is linked to localized, in situ immunological processes [9,10]. This discrepancy could explain the lack of substantial relationships between tubulointerstitial nephritis and measures of systemic disease activity.

This study suggested significant differences in serum C3, C4, and anti-dsDNA antibody markers of systemic autoimmunity between the proliferative GN and nonproliferative GN groups. Conversely, there was no noticeable difference in these values according to the severity of TI (p>0.05). These results imply that, regardless of glomerular involvement and/or systemic autoimmunity, TI assessment represents a significant pathogenic process that may be limited to the renal tissue. This study's external reliability and generalizability are supported by the agreement of its findings with earlier research [1,7,8].

In a retrospective study, tubular proteinuria was found to result from the unimpeded filtration of low-molecular-weight proteins through the glomerulus due to impaired reabsorption of the proximal tubule owing to proximal tubular injury [27]. Even though there is evidence that urinary tubular biomarkers may be used to determine mortality risk, concerns over the cost-effectiveness of measuring specific tubular proteins pose a significant obstacle to their widespread clinical adoption. The significant linear connection between uNAP and all-cause mortality underlines its potential as an independent predictor of mortality risk, perhaps providing more sensitivity than established measurements such as uPCR or uACR. Therefore, simultaneous uPCR and uACR measurement is reasonably priced and offers measurable information about the degree of tubular proteinuria [27].

According to recent research, which employed the same criteria of uNAP as this study, a substantial correlation between uNAP and tubulointerstitial inflammation in lupus nephritis patients was found [8,20]. Notably, a significant correlation has been shown between high uNAP levels and a poor response to immunosuppressive therapies [8,20]. NAP may be useful for evaluating long-term renal effects in lupus nephritis cases since early response to induction therapy is crucial for long-term renal outcomes [5,22].

In this study, the cutoff value for uPCR-ACR was established, which stood at 887. This specific threshold is linked to a sensitivity of 96.7% and a specificity of 76.0%. Although previous studies looked at uPCR-uACR and its association with TI, none reported a threshold or cutoff value that might be useful for clinicians to suspect more severe TI involvement in the future based on this simple diagnostic test.

Current guidelines do not yet support the routine use of urine albumin-to-creatinine ratio (uACR) for lupus nephritis monitoring, although they recommend serial urine protein-to-creatinine ratio (uPCR) measures in the follow-up of lupus nephritis patients [8]. This study found a significant association between the degree of tubulointerstitial inflammation and non-albumin proteinuria, which is classified as uPCR-uACR. Thus, concurrently monitoring urine albumin and total protein levels may be useful for assessing the severity of TI and predicting long-term renal prognosis in patients with lupus nephritis.

## Conclusions

This study concludes that the severity of TI is highly associated with non-albumin proteinuria, as determined by the difference between uPCR and uACR in lupus nephritis. Conventional parameters cannot assess TI severity. Moreover, kidney biopsy is not convenient to do serially due to significant comorbidity. In this context, measuring non-albumin proteinuria seems to be clinically significant as a non-invasive, affordable, and accurate parameter to determine the severity of TI. Therefore, non-albumin proteinuria can be monitored serially by clinicians for the assessment of the severity of TI in lupus nephritis patients.
